# Effectiveness of the Family Portal Function on the Lilly Connected Care Program (LCCP) for Patients With Type 2 Diabetes: Retrospective Cohort Study With Propensity Score Matching

**DOI:** 10.2196/25122

**Published:** 2021-02-05

**Authors:** Yiyu Zhang, Chaoyuan Liu, Shuoming Luo, Jin Huang, Yuxin Yang, Xiao Ma, Xia Li, Zhiguang Zhou

**Affiliations:** 1 National Clinical Research Center for Metabolic Diseases, Key Laboratory of Diabetes Immunology of Ministry of Education, Department of Metabolism and Endocrinology, The Second Xiangya Hospital Central South University Changsha China; 2 Department of Endocrinology Changsha Central Hospital Changsha China; 3 Department of Oncology The Second Xiangya Hospital Central South University Changsha China; 4 Lilly Suzhou Pharmaceutical Company Suzhou China

**Keywords:** diabetes management, diabetes mellitus, family support, mobile app, app, self-management, support, online portal, diabetes, cohort

## Abstract

**Background:**

Diabetes is a major health concern worldwide. Family member engagement in diabetes self-management education programs can improve patients’ diabetes management. However, there is limited evidence that the family portal on diabetes management apps is effective in the glycemic control of patients with diabetes.

**Objective:**

We aimed to evaluate the effectiveness of family support through the family portal function on Lilly Connected Care Program (LCCP) platform.

**Methods:**

This retrospective cohort study included patients with type 2 diabetes recruited to the LCCP platform from September 1, 2018, to August 31, 2019. Propensity score matching was used to match family (group A) and non–family (group B) portal use groups with similar baseline characteristics. The patients were followed up with for 12 weeks. The main objectives were differences in mean fasting blood glucose, proportion of patients achieving fasting blood glucose target <7mmol/L, mean postprandial blood glucose, proportion of patients achieving postprandial blood glucose target <10mmol/L, proportion of patients achieving both fasting blood glucose <7mmol/L and postprandial blood glucose <10mmol/L, self-monitoring of blood glucose frequency at week 12 and the number of diabetes education courses patients completed during the 12 weeks. Moreover, logistic regression analysis was used to explore the baseline factors which may be associated with the use of family portal, and odds ratios with 95% confidence intervals were calculated.

**Results:**

A total of 6582 adult patients (aged ≥18 years) with type 2 diabetes who were receiving insulin therapy were enrolled in the study. Overall, 6.1% (402/6582) of the patients chose to engage their family members to use the family portal. Two groups of 394 patients were well-matched regarding baseline characteristics. After matching, mean fasting blood glucose and postprandial blood glucose at week 12 were significantly lower in group A than in group B (fasting blood glucose: 7.12 mmol/L, SD 1.70 vs 7.42 mmol/L, SD 1.88, respectively, *P*=.02; postprandial blood glucose: 8.56 mmol/L, SD 2.51 vs 9.10 mmol/L, SD 2.69, respectively, *P*=.002). When comparing group A to group B, the proportion of patients achieving both fasting blood glucose <7mmol and postprandial blood glucose <10mmol/L at week 12 (46.8% vs 39.4%, respectively, *P*=.04), self-monitoring of blood glucose frequency at week 12 (8.92 times per week, SD 6.77 vs 8.02 times per week, SD 5.97, respectively, *P*=.05) and number of diabetes education courses completed in 12 weeks (23.00, IQR9.00-38.00 vs 15.00, IQR 4.00-36.00, respectively, *P*<.001) was higher. Additionally, multivariate logistic regression analysis showed that higher age (OR=0.987, 95% CI 0.978-0.996, *P*=.006) and higher baseline fasting blood glucose (OR=0.914, 95% CI 0.859-0.972, *P*=.004) were correlated with less use of the family portal function, while increased baseline self-monitoring of blood glucose frequency (OR=1.022, 95% CI 1.012-1.032], *P*<.001) as well as increased education courses (OR=1.026, 95% CI 1.015-1.036, *P*<.001) were associated with more use of the family portal function.

**Conclusions:**

Family support through the LCCP family portal is effective for glycemic control and self-management behavior improvement in type 2 diabetes patients.

## Introduction

### Background

Diabetes is a major health concern worldwide [1,2]. In 2013, the prevalence of diabetes in China was 10.4%, representing more than 100 million adults living with diabetes [3]. However, only 39.7% of those treated had ideal glycemic control [4]. Poor glycemic control leads to various complications [5] and brings heavy economic and social burden to the world. The global cost of diabetes was estimated to be up to US $1.31 trillion in 2015 [6].

Diabetes treatment depends on life-long self-management behaviors including maintaining a healthy diet, engaging in regular physical activity, self-monitoring blood glucose, and adhering to prescribed mediation routines [7], which are often inadequate and unsustainable [8]. Diabetes self-management education is critical for patients’ self-management behaviors [9]. Because some patients have poor understanding and cooperation, interventions aiming to improve self-management behaviors are not always effectively implemented [10]. Another possible reason for poor self-management behaviors is the lack of diabetes-specific support from social networks, especially family members [11].

Family members can provide patients with financial support, emotional support, supervision and reminders of self-management behaviors, and instrumental support such as administering insulin injections [12,13]. The Chinese culture attaches great importance to the relationship between family members [14]. Confucianism beliefs recognize that everyone is naturally born to, grows up in, and is taken care of within a family [15]. This cultural context makes it so that family members play a key role in diabetes management. Family member engagement in diabetes self-management education programs can improve patients’ self-management behaviors, quality of life, and glycemic control [16-18]. Studies have also shown that family-model diabetes self-management education is superior to conventional diabetes education that only involves patients [19,20]. However, family members, especially young members, cannot always participate in diabetes management programs that require onsite visits for long periods of time [11].

Mobile apps can receive and transmit information at any time and any place. With the popularity of smartphones, mobile apps represent a promising technology for supporting diabetes management [21]. Many diabetes management apps provide interhuman communications, blood sugar records, diabetes education, and more [22]. Some diabetes management apps have family portals through which family members can (1) view the blood glucose records of the patient, (2) provide support in diabetes management, and (3) receive diabetes self-management education [23,24]. However, there is limited evidence that the family portals of diabetes management apps are effective in the glycemic control of patients with diabetes, and the characteristics of patients who invite their family members to engage in diabetes management through family portals on diabetes management apps are not very clear.

The Lilly Connected Care Program (LCCP) is a national diabetes care and support program delivered by the LCCP official account on China's largest social app, WeChat. There are more than 60 diabetes education courses created by experts in accordance with the standards of medical care for type 2 diabetes mellitus in China on the LCCP platform. Insulin therapy is the cornerstone of treatment for patients with type 2 diabetes who fail to obtain target glycemic control with oral hypoglycemic agents or for patients who are contraindicated for oral hypoglycemic agents [25]. Our previous study [26] has found that LCCP app-based diabetes education is effective for glycemic control and can improve self-monitoring of blood glucose behavior in patients with type 2 diabetes receiving insulin therapy. Through the family portal function on the LCCP platform, patients can choose to engage family members in their diabetes management.

### Objective

The aim of this study is to evaluate the effectiveness of family support through the family portal on the LCCP platform for patients using insulin therapy.

## Methods

### Research Design and Samples

This retrospective cohort study included patients with diabetes recruited to the LCCP platform from September 1, 2018 to August 31, 2019. Patients with diabetes receiving insulin treatment were encouraged by their doctors to register on the LCCP platform. Patient demographic and disease information, including age, gender, education level, type of diabetes, insulin regimen, and duration of diabetes, were collected after provision of written informed consent. The patients in this study were followed up with for 12 weeks. Eligible samples were adult type 2 diabetes patients (aged ≥18 years) with fasting blood glucose and postprandial blood glucose levels recorded using the LCCP platform at least once a week at week 1 and week 12. Patients with type 1 diabetes, patients aged <18 years, and those with missing data on gender, age, education level, type of diabetes, and diabetes duration were excluded from the study.

### Intervention

All the patients recruited to the LCCP platform were able to record their blood glucose and take more than 60 diabetes education courses on the LCCP platform. The patients were all informed of the function of the LCCP family portal when recruited and voluntarily chose to engage their family members to use the family portal connected to their private LCCP account. Patient self-monitoring of blood glucose data were automatically sent to the family portal in real time. Family members using the family portal were able to view the patients’ blood glucose records and diabetes education course learning records, take the diabetes education courses, and participate in 2-way communications with the patients through the family portal.

### Outcome Measurements

Patients recruited to the LCCP platform were all provided with a free intelligent glucometer. Self-monitoring of finger-prick capillary blood glucose was assessed according to the glucose dehydrogenase method using a glucometer (Bionime Biotechnology [Ping Tan] Co, Ltd, Fuzhou City, China). The patients were trained to test their fasting blood glucose and postprandial blood glucose correctly to reduce subject bias. Patient self-monitoring of blood glucose data were automatically transmitted to the LCCP platform through mobile signals. The coefficient of variation of the measurement was below 5%, and the accuracy was in accordance with ISO 15197:2013 [27]. The patients were divided into 2 groups: group A (the family portal use group) and group B (the non–family portal use group). We defined patient baseline fasting blood glucose and postprandial blood glucose as the mean fasting blood glucose and mean postprandial blood glucose at the first week after recruitment. The primary outcomes were differences between group A and group B in mean fasting blood glucose, mean postprandial blood glucose, proportion of patients achieving fasting blood glucose <7 mmol/L, proportion of patients achieving postprandial blood glucose <10 mmol/L, proportion of patients achieving both fasting blood glucose <7 mmol/L and postprandial blood glucose <10 mmol/L, frequency of self-monitoring of blood glucose at week 12, and difference in the number of diabetes education courses that the patients completed in the 12 weeks of the study.

### Ethics

All patients provided written informed consent when they were recruited to the LCCP platform. The study conformed to Declaration of Helsinki principles and was approved by the ethics committee of the Second Xiangya Hospital.

### Propensity Score Matching

Propensity score matching was used to match group A (the family portal use group) and group B (the non–family portal use group) with similar baseline characteristics. The propensity score was calculated using a multivariable logistic regression model, with the use of family portal as the dependent variable and potential confounding factors as covariates, including age, gender, education level, insulin regimen, duration of diabetes, baseline fasting blood glucose, baseline postprandial blood glucose, and baseline self-monitoring of blood glucose frequency. Because we defined patient baseline blood glucose as the mean blood glucose level in the first week, and as our previous study found that taking the diabetes education courses on the LCCP platform can influence patients’ glycemic control, we also included the number of diabetes education courses that the patients completed in the first week as a covariate in the logistic regression model. Matching was performed on a ratio of 1:1 using a nearest-neighbor algorithm with no replacement (Greedy 8-1 digit match algorithm), with a caliper width of 0.02. Once a match was made, patients were not reconsidered for further matching. Standardized mean differences were used to assess comparability of the 2 groups on each confounding variable after matching. A standardized mean difference of less than 10.0% for a given covariate indicates a balance between groups [28,29].

### Statistics

Continuous variables with normal or near-normal distributions are presented as means with standard deviations. Variables with nonnormal distributions are presented as medians with IQRs. Categorical variables are presented as the frequency (number of cases, n) and percentage (%) of total study patients. Chi square test was used for categorical variables; t test and paired t test were used for continuous variables with normal distribution before and after matching, respectively; Wilcoxon rank-sum test and Wilcoxon signed-rank test were used for continuous variables with nonnormal distribution before and after matching, respectively. These tests were used to compare baseline characteristics and outcome measurements at week 12 between the 2 groups. The baseline factors which may have been associated with family portal use were explored using univariate and multivariate logistic regression models, and odds ratios with 95% confidence intervals were calculated. Statistical analysis was performed using SAS 9.4 software (SAS Institute, North Carolina, USA) via SAS Enterprise Guide version 7.1. *P* values ≤.05 were considered statistically significant.

## Results

### Patient Characteristics at Baseline

**Figure 1:**
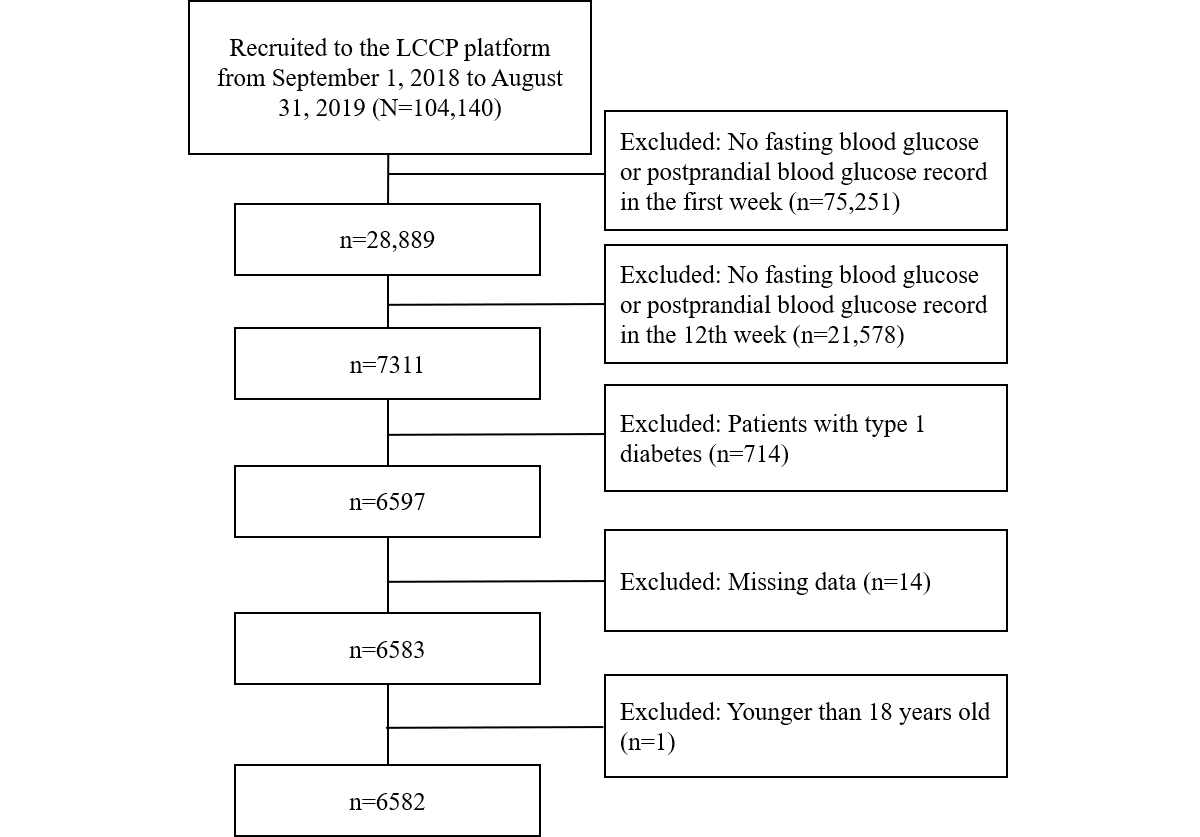
Patient inclusion flow diagram.

A total of 6582 adult patients (aged ≥18 years) with type 2 diabetes who were receiving insulin therapy were enrolled in the study. The samples were recruited from 31 provinces across China. Figure 1 shows the patient inclusion flow diagram. Among the total participants, 56.3% (3705/6582) were male. The median age was 53.07 years (IQR 43.81-61.10 years), and the median disease duration was 27.67 months (IQR 1.23-114.17 months). The mean fasting blood glucose at baseline was 7.76 (SD 2.21) mmol/L, and the mean postprandial blood glucose was 9.51 (SD 2.88) mmol/L. The mean frequency of self-monitoring of blood glucose at baseline was 13.63 (SD 9.18) times per week (see Table 1).

**Table 1:** Patient characteristics at baseline (N=6582).

Variable		Value^a^
**Gender, n (%)**		
	Male	3705 (56.3)
	Female	2877 (43.7)
**Education, n (%)**		
	Junior middle school or below	1830 (27.8)
	High school	2165 (32.9)
	College or above	2587 (39.9)
Age, mean years (IQR)		53.07 (43.81-61.10)
Baseline fasting blood glucose, mmol/L (SD)		7.76 (2.21)
Baseline postprandial blood glucose, mmol/L (SD)		9.51 (2.88)
Duration of diabetes, months (IQR)		27.67 (1.23-114.17)
Baseline self-monitoring of blood glucose frequency, times per week		13.63 (SD 9.18)
**Insulin regimen, n (%)**		
	Premixed insulin	5240 (79.6)
	Fast-acting insulin (with/without long-acting insulin)	1342 (20.4)
Baseline education courses, n (IQR)		2.0 (1.0-5.0)

^a^Continuous variables are presented as means with standard deviations or medians with IQRs, and categorical variables are presented as n (%).

### Comparisons of Baseline Characteristics Between the Family Portal Use Group and Non–Family Portal Use Group After Matching

Overall, 6.1% (402/6582) of the patients chose to engage their family members to use the family portal. With the use of propensity score matching, 394 family portal use patients were matched with 394 non–family portal use patients. After propensity score matching, gender (*P*=.61), age (*P*=.38), education level (*P*=.54), duration of diabetes (*P*=.49), insulin regimen (*P*=.47), fasting blood glucose (*P*=.51), postprandial blood glucose (*P*=.34), self-monitoring of blood glucose frequency (*P*=.75), and the number of diabetes education courses completed (*P*=.44) showed no significant differences at baseline between the 2 groups, and the standardized mean differences were <10.0% for all variables, indicating a good balance between the 2 groups at baseline (see Table 2).

**Table 2:** Comparisons of baseline characteristics between group A (the family portal use group) and group B (the non–family portal use group) after matching.

Variable^a^		Group A (n=394)	Group B (n=394)	Standardized mean difference, %	*P* value	
**Gender, n (%)**						.61
	Male	232 (58.9)	225 (57.1)	3.59	N/A^b^	
	Female	162 (41.1)	169 (42.9)	3.59	N/A	
**Education, n (%)**						.54
	Junior middle school or below	95 (24.1)	82 (20.8)	7.91	N/A	
	High school	126 (32.0)	133 (33.8)	3.79	N/A	
	College or above	173 (43.9)	179 (45.4)	3.06	N/A	
Age, years (IQR)		50.16 (42.34-58.46)	50.69 (42.20-60.02)	7.62	.38	
Baseline fasting blood glucose, mmol/L (SD)		7.44 (1.91)	7.50 (1.88)	2.96	.51	
Baseline postprandial blood glucose, mmol/L (SD)		9.40 (2.82)	9.55 (2.84)	5.25	.34	
Duration of diabetes, months (IQR)		18.84 (1.10-100.27)	25.92 (1.33-108.20)	4.72	.49	
Baseline self-monitoring of blood glucose frequency, times per week (SD)		16.20 (10.30)	16.33 (10.22)	1.29	.75	
**Insulin regimen, n (%)**						.47
	Premixed insulin	324 (82.2)	316 (80.2)	5.2	N/A	
	Fast-acting insulin (with/without long-acting insulin)	70 (17.8)	78 (19.8)	5.2	N/A	
Baseline education courses, n (IQR)		3.0 (1.0-9.0)	2.0 (1.0-8.0)	1.83	.44	

^a^Continuous variables are presented as means with standard deviations or medians with IQRs, and categorical variables are presented as n (%).

^b^N/A: not applicable.

### Effectiveness of LCCP Family Portal

Before matching, compared with the non–family portal use group, the family portal use group had lower fasting blood glucose (7.10 mmol/L, SD 1.70 vs 7.48 mmol/L, SD 2.03, *P*<.001); lower postprandial blood glucose (8.57 mmol/L, SD 2.81 vs 8.97 mmol/L, SD 2.78, *P*=.002); a higher proportion of patients achieving fasting blood glucose target <7mmol/L (52.7% vs 47.4%, *P*=.04), postprandial blood glucose target <10mmol/L (77.6% vs 71.5%, *P*=.009), and both fasting blood glucose <7mmol/L and postprandial blood glucose <10mmol/L (47.5% vs 40.7%, *P*=.008); and higher self-monitoring of blood glucose frequency (8.94 times per week, SD 6.72 vs 8.01 times per week, SD 5.85, *P*=.007) at week 12 and a higher number of diabetes education courses completed in the entire 12 weeks (23.5 courses, IQR 10.0-38.0 vs 13.0 courses, IQR 4.0-33.0, *P*<.001). After controlling for baseline potential confounders using propensity score matching, fasting blood glucose and postprandial blood glucose at week 12 were still significantly lower in the family portal use group than in the non–family portal use group (fasting blood glucose: 7.12 mmol/L, SD 1.70 vs 7.42 mmol/L, SD 1.88, respectively, *P*=.02; postprandial blood glucose: 8.56 mmol/L, SD 2.51, vs 9.10 mmol/L, SD 2.69, respectively, *P*=.002). The proportion of family use group patients achieving both fasting blood glucose <7mmol and postprandial blood glucose <10mmol/L was higher than that of non–family use group patients (46.8% vs 39.4%, respectively, *P*=.04), as was the self-monitoring of blood glucose frequency at week 12 (8.92 times per week, SD 6.77, vs 8.02 times per week, SD 5.92, respectively, *P*=.050) and number of diabetes education courses completed in 12 weeks (23.0 courses, IQR 9.0-38.0, vs 15.00 courses, IQR 4.0-36.0, respectively, *P*<.001) (see Table 3).

**Table 3:** Comparison of the outcomes at the 12th week between group A (the family portal use group) and group B (the non–family portal use group) before and after matching.

Outcome^a^	Before matching				After matching		
	Group A (n=402)	Group B (n=6180)	*P* value	Group A (n=394)		Group B (n=394)	*P* value
Fasting blood glucose (12th week), mmol/L (SD)	7.10 (1.70)	7.48 (2.03)	<.001	7.12 (1.70)		7.42 (1.88)	.02
Fasting blood glucose <7 mmol/L (12th week), n (%)	212 (52.7%)	2931 (47.4%)	.04	204 (51.9%)		178 (45.3%)	.07
Postprandial blood glucose (12th week), mmol/L (SD)	8.57 (2.81)	8.97 (2.78)	.002	8.56 (2.51)		9.10 (2.69)	.002
Postprandial blood glucose <10 mmol/L (12th week), n (%)	312 (77.6%)	4417 (71.5%)	.009	304 (77.4%)		287 (73.0%)	.19
Fasting blood glucose <7 mmol/L and postprandial blood glucose <10 mmol/L (12th week), n (%)	191 (47.5%)	2513 (40.7%)	.008	184 (46.8%)		155 (39.4%)	.04
Self-monitoring of blood glucose frequency (12th week), times per week (SD)	8.94 (6.72)	8.01 (5.85)	.007	8.92 (6.77)		8.02 (5.97)	.05
Education courses, n (IQR)	23.5 (10.0-38.0)	13 (4.0-33.0)	<.001	23 (9.0-38.0)		15 (4.0-36.0)	<.001

^a^Continuous variables are presented as means with standard deviations or medians with IQRs, and categorical variables are presented as n (%).

### Analysis of Baseline Factors Associated With the Use of Family Portal

To further investigate the baseline factors correlating with the use of family portal, we performed univariate and multivariate model regression analyses. According to the univariate model regression analysis, junior middle school education or below (OR=0.754, *P*=.03), increased age (OR=0.982, *P*<.001), higher baseline fasting blood glucose (OR=0.92, *P*=.002), and longer duration of diabetes (OR=0.999, *P*=.03) were associated with a smaller number of patients using the family portal function, while increased self-monitoring of blood glucose frequency (OR=1.031, *P*<.001) and increased education courses (OR=1.032, *P*<.001) were associated with elevated number of patients using the family portal function (see Table 4). Moreover, multivariate logistic regression analysis showed that higher age (OR=0.987, *P*=.006) and higher baseline fasting blood glucose (OR=0.914, *P*=.004) were independent factors correlating with less use of the family portal function, while increased self-monitoring of blood glucose frequency (OR=1.022, *P*<.001) as well as increased education courses (OR=1.026, *P*<.001) were independent predictive factors for greater use of the family portal function (see Table 4).

**Table 4:** Baseline factors associated with the use of family portal according to logistic regression analysis.

Variable			Univariate model OR (95% CI)		*P* value		Multivariate model OR (95% CI)		*P* value
**Gender**									
	Male	1.148 (0.935-1.410)		.19		1.075 (0.873-1.324)		.50	
	Female	Reference		N/A^a^		Reference		N/A	
**Education**									
	Junior middle school or below	0.754 (0.584-0.974)		.03		0.863 (0.664-1.121)		.27	
	College or above	0.863 (0.682-1.091)		.22		0.938 (0.738-1.191)		.60	
	High school	Reference		N/A		Reference		N/A	
Age			0.982 (0.974-0.990)		<.001		0.987 (0.978-0.996)		.006
Baseline fasting blood glucose			0.920 (0.873-0.969)		.002		0.914 (0.859-0.972)		.004
Baseline postprandial blood glucose			0.985 (0.950-1.021)		.40		1.039 (0.995-1.085)		.08
Duration of diabetes (months)			0.999 (0.997-1.000)		.03		1.000 (0.999-1.001)		.84
Baseline self-monitoring of blood glucose frequency			1.031 (1.022-1.041)		<.001		1.022 (1.012-1.032)		<.001
**Insulin regimen**									
	Premixed insulin	1.016 (0.790-1.306)		.90		1.089 (0.844-1.407)		.51	
	Fast-acting insulin (with/without long-acting insulin)	Reference		N/A		Reference		N/A	
Baseline education courses			1.032 (1.022-1.042)		<.001		1.026 (1.015-1.036)		<.001

^a^NA: not applicable.

## Discussion

### Principal Findings

We evaluated the effectiveness of family support through the family portal on the LCCP platform for patients receiving insulin therapy and found that the family portal on the LCCP platform was effective for glycemic control. Before propensity score matching, the family portal use group had lower fasting blood glucose, lower postprandial blood glucose, a higher percentage of participants who achieved fasting blood glucose and postprandial blood glucose control targets, separately, and a higher percentage of participants who achieved both fasting blood glucose and postprandial blood glucose control targets at the same time after 12 weeks of intervention compared with the non–family portal used group. However, those choosing to engage their family members in the use of the LCCP family portal might be more active in glycemic control. Thus, we controlled for baseline potential confounders by propensity score matching, including baseline blood glucose, age, gender, duration of diabetes, insulin regimen, and self-monitoring of blood glucose frequency. After matching, the two cohorts were well-matched regarding baseline characteristics, and both fasting blood glucose and postprandial blood glucose at week 12 of the family portal use group were lower and more patients were achieving blood glucose control targets of both fasting blood glucose and postprandial blood glucose at the same time at week 12 as compared to the non–family portal use group. These data indicate that the family portal function on LCCP platform contributes to the glycemic control of diabetes patients receiving insulin therapy.

Studies have shown that family members’ support is related to patients’ self-management behavior and glycemic control [30]. In our study, family members can take diabetes education courses through the family portal on the LCCP platform. Obtaining more diabetes management knowledge makes it easier for family members to provide regimen-related decision-making and problem-solving support, and family members with more knowledge about diabetes tend to perform more diabetes-specific supportive behaviors [31], including support in meal planning and encouragement of regular physical activity. In addition, we found that the family portal use group took more diabetes education courses on the LCCP platform and had higher self-monitoring of blood glucose frequency at 12 weeks before and after propensity-score matching compared to the non–family portal use group. These could be the possible mechanisms by which the use of the family portal led to glycemic control improvement. Many studies have suggested that diabetes education can improve glycemic control and self-management behaviors of patients [32-35]. Family members can monitor the behavior of patients’ diabetes education course learning through the family portal, and the study by McElfish et al [19] has revealed that family-model diabetes self-management education shows better effects than standard diabetes self-management education [19]. The family-model can increase the time of exposure to diabetes self-management education, and increased time of exposure to leads to improved glycemic control in patients [20]. The diabetes education courses on the LCCP platform cover patients’ self-care behaviors according to the American Association of Diabetes Educators 7 Standard of Care. Our previous study has also found that patients taking more diabetes education courses on the LCCP platform had better glycemic control [26].

Self-monitoring of blood glucose is an important part of diabetes self-management in patients receiving insulin therapy; it is useful for patients for adjusting insulin dosage and guiding nutrition therapy and physical activity [36]. The family portal use group had a higher frequency of self-monitoring of blood glucose, possibly because they took more diabetes education courses on the LCCP platform and received telemonitoring from their family members through the family portal. Taking more diabetes education courses could have increased their awareness of the importance of self-monitoring of blood glucose. Previous studies have revealed that diabetes self-management education can improve patients’ self-management behaviors, including self-monitoring of blood glucose [9,33]. However, some patients may not attach much importance to self-monitoring of blood glucose [37]. Family members can provide real-time telemonitoring and may be able to remind patients to self-monitor their blood glucose behaviors. Aikens’ study [38] has found that integrating support persons into diabetes telemonitoring can improve patients’ self-management and medication adherence. This may be particularly helpful for those living out of home and for long-distance family members [39].

Some mobile health solutions now allow patients to invite family members as support persons in disease management. However, few studies have examined characteristics of patients who choose to engage a support person in their healthcare [39]. Our study found that patients with decreased age, lower baseline fasting blood glucose level, higher self-monitoring of blood glucose frequency, and more completed education courses were more likely to use the family portal. Previous studies [40-42] have found that younger patients have a higher usage of diabetes management apps. One study using text messaging to engage family members in diabetes self-management support also found that participants who invited a support person were younger than those who did not [39]. Young patients may be more receptive to new technologies and, as such, may be more inclined to use new technologies with their families in order to manage their diabetes. Otherwise, we observed that lower baseline fasting blood glucose levels were associated with the tendency for participants to invite family members to use the family portal. The reasons for this are not quite clear. A similar negative association of baseline fasting blood glucose with use of mobile app is also observed in another study [43], which infers that the patients with higher fasting blood glucose levels were more reticent to share the high values with their family members, and this fact may explain our results. Moreover, patients with higher self-monitoring of blood glucose frequency may have more initiative in their disease management and raised awareness of their disease after their frequent monitoring; therefore, they may tend to invite family members to participate in their disease management. Additionally, patients who have completed more education courses have obtained more knowledge on diabetes as well as a fuller understanding of the family portal in the LCCP platform compared with those with less education courses; thus, they may be willing to invite their family members to participate in their disease management.

The proportion of patients who invited their family members to participate using the family portal was very low. The possible reasons are as follows: first, although patients were all informed of the function of LCCP family portal, many family members still did not know they were able to view the patients’ blood glucose records and diabetes education course learning records by using the family portal connected to their private LCCP account; second, some patients may be unwilling to be monitored by their family members or to bother their family members too much. We may further investigate the possible reasons for the very low usage of the family portal and explore the factors that influence family members to join the family portal on the LCCP platform to improve the app’s design.

Although randomized control trials are generally considered to show the most reliable evidence for medical research, they often fail to reflect real-world clinical practice [44,45]. Our study was based on real-world data, and we adjusted the potential confounding factors at baseline by propensity-score matching. Thus, our study can be used as evidence for the efficacy of the family portal function in addition to evidence gathered through randomized controlled trials.

### Limitations

Our study has several limitations. First, our observation period was short. The long-term effect of the LCCP family portal needs further investigation, and future studies could use glycosylated hemoglobin as an indicator of blood glucose control. Second, we only investigated the characteristics of patients; the characteristics of family members (such as age, education level, and history of diabetes) were not investigated. In addition, we did not assess the degree of family members’ engagement in the LCCP family portal, despite the fact that the degree of engagement could directly influence the intervention effects.

### Conclusions

The LCCP family portal is effective for glycemic control and self-management behavior improvement in patients with type 2 diabetes receiving insulin therapy. It is convenient and timesaving for family members to use app-based family portals to provide diabetes management support. The family portal has great potential to be used as a supplement to traditional social support for diabetes management.

## Abbreviations

LCCP: Lilly Connected Care Program
